# New Appliances and Things Medical

**Published:** 1910-04-16

**Authors:** 


### NEW APPLIANCES AND THINGS MEDICAL.

[We shall be glad to receive at our Office, 28 & 29 Southampton Street, Strand, London, W.C., from the manufacturers, specimens of all new preparations and appliances.]

### THIOCOL.

Thiocol, a preparation samples of which have been sent us, is described as a potassium salt of guaiacol sulphate, and is designed for use in tuberculosis, pulmonary affections, pneumonia, bronchitis, and whooping cough—rather a wide range of application. The action claimed for it is "essentially bactericidal," apparently not merely that of an intestinal disinfectant. The dose is 8 grains, three to six times a day, and may be increased to 15 grains. Supplied by the Hoffmann-La Roche Chemical Works, Idol Lane, London.

### THE SYNTAM INDEXING AND FILING SYSTEM.

Messrs. E. R. Norman, whose system of filing we recently noticed in this column, write to state that the name of their system was incorrectly given in the notice as "The Syntax System," whereas it should have been "The Syntam System." The Syntam is an all-British system, and has no connection with any American patent. Messrs. E. R. Norman and Co. are the sole proprietors, and will be glad to forward their illustrated booklet, giving full details of the methods used, on application.

### TETRA PATENT SURGICAL DRESSINGS.

(Hospitals and General Contracts Co., Ltd., 33 and 35 Mortimer Street, London, W.)

These surgical dressings are well worth a trial, since they are simple, efficient, and durable. The "Tetra" gauze pads, which are supplied in a dozen sizes, are made of fourfold gauze with selvedge borders, so that the pad is practically untearable, while it is easily cut with a pair of scissors. This pad is supplied in quantities of one hundred or more in the smaller, and in packets of ten or more of the larger sizes at very cheap rates. The twofold gauze pads and the gauze rolls, all with selvedging, are similar in make. We have tested the gauze and have found it an excellent dressing, a good absorber, and one which is easily removed from septic wounds. The firm supplies a large number of specialities both in instruments and in dressings. Their illustrated catalogues are sent post free on application.

### THE RUBBER BOOM.

Rubber is booming. Not only is the Stock Exchange affected, but raw rubber has been sold for record prices—considerably more than 100 per cent. above the normal. This seriously affects those trades whose staple products require the consumption of large quantities of rubber, and any margin of profit is more than swallowed up by the serious advances of the raw materials. The manufacturer is faced with a problem, a very serious problem. Shall he make his goods of a lower standard of quality, or shall be, in face of competition, advance his prices to the public? The makers of Redfern's Navy Rubber Heels are confident that the public as a whole prefer the best quality article even if it costs a trifle more. They have decided to continue to make their rubber heels of the standard quality which has gained for them the good repute of the whole world. Quality has made their market—quality shall retain it. So they accept the alternative and ask the public to pay a little more to enable them to keep this standard of quality. In future Redfern's Navy Pads will be sold at: men's, 7½d. per pair; ladies' and children's, 5d. per pair. They are obtainable at all boot dealers and repairers.

### A PATENT INVALID LIFTER.

(Skeffington's Patent Invalid Lifter, Temporary Address, 49 Ulundi Road, Blackheath, S.E.)

At the Ideal Home Exhibition, now open at Olympia, can be seen a stall where a good model of an invalid lifter is exhibited in working order. This is the "Skeffington," a new model, which is eminently adapted for hospital use. In many cases the trouble and inconvenience to patients and nurses alike caused by the old-fashioned methods of turning or lifting invalids in bed are easily obviated by the employment of a simple lifting apparatus. In all the large Continental hospitals a lifter is to be seen in every ward, and hitherto we have seen no better model than that used in the Moabite Hospital at Berlin, the invention of the superintendent. The Skeffington lifter, however, is in every way a useful and excellent contrivance. It can be easily clamped to any bed, consisting as it does mainly of a roller and lifting sheet, and it is operated easily and simply by a few turns of the handle. The patient is lifted clear off the mattress, so that the bed can be made underneath him. No strength is necessary for turning the handle, and a child can use the lifter. The model is so simply constructed and of such durable material that it ought to last a lifetime, and one instrument will serve a whole ward or series of wards. It is an appliance which we should like to see in every institution.

THE HOSPITAL. April 16, 1910.

